# Line Position-Dependent Effect in Line-by-Line Inscribed Fiber Bragg Gratings

**DOI:** 10.3390/s21217231

**Published:** 2021-10-30

**Authors:** Hongye Li, Xiaofan Zhao, Binyu Rao, Meng Wang, Baiyi Wu, Zefeng Wang

**Affiliations:** 1 College of Advanced Interdisciplinary Studies, National University of Defense Technology, Changsha 410073, China; lihongye@nudt.edu.cn (H.L.); zhaoxiaofan_zxf@nudt.edu.cn (X.Z.); raobinyu@nudt.edu.cn (B.R.); wangmeng@nudt.edu.cn (M.W.); wubaiyi@nudt.edu.cn (B.W.); 2 State Key Laboratory of Pulsed Power Laser Technology, Changsha 410073, China; 3 Hunan Provincial Key Laboratory of High Energy Laser Technology, Changsha 410073, China

**Keywords:** femtosecond laser, fiber Bragg gratings, cladding mode, apodization

## Abstract

Line-by-line direct writing by femtosecond laser has been proved to be a simple and effective method for the fabrication of low-loss fiber Bragg gratings (FBGs), and is more flexible compared with the traditional ultraviolet exposure method. In this paper, the line-position-dependent characteristics of cladding modes coupling in line-by-line FBGs have been studied, to the best of our knowledge, for the first time. Both theoretical and experimental results show that off-center inscribing could compress the bandwidth of the Bragg resonance and excite more abundant cladding mode coupling, in which the core-guided fundamental mode would couple to the cladding-guided LP0n and LP1n simultaneously. By aligning the line positions across the core region, the first apodized line-by-line FBG was achieved. This work enriches the theories of line-by-line FBGs and provides an inscription guidance to meet different application requirements.

## 1. Introduction

The femtosecond laser direct writing technique is a nice choice for fabricating fiber Bragg gratings (FBGs). Compared with the phase mask technique [1,2], the direct writing technique [3,4] is much more flexible and prevents the limitation of phase masks. The point-by-point (PBP) method is the most common way of directly writing FBGs [5,6,7]; however, point-by-point fiber Bragg gratings (PBP-FBGs) are often with high insertion loss [8], which limits their applications in many fields, such as fiber lasers. Another direct writing method, named line-by-line (LBL), has been proved to be effective for inscribing lower insertion loss and polarization-dependent FBGs. This method was firstly proposed by Zhou et al in 2010 [9], and the relevant studies have been thriving in the past decade.

Line-by-line fiber Bragg gratings (LBL-FBGs) have already been utilized in fiber sensors and fiber lasers. Chah et al. proposed that higher order LBL-FBG inscribed by a long-working distance objective without matching oil was highly birefringent, meaning this kind of LBL-FBG could play an important role in transverse force measurements [10]. Huang et al. reported that a phase-shifted FBG fabricated by the LBL inscription could be applied in a fiber torsion sensor for its strong birefringence [11]. Another kind of phase-shifted FBG inscribed on both sides of a fiber taper with the LBL technique was proved to be effective in axial strain detection [12]. The LBL technique was also utilized to inscribe a random-distributed grating array, which played the role of random feedback in an Er-doped random fiber laser [13]. Yang et al. put forward a novel LBL-FBG-based optical fiber tag to distinguish data transmission cables [14]. Besides, due to a larger overlap between refractive index modulation and mode profile compared with PBP-FBGs, the LBL technique was suitable for large core FBG inscription. For example, Xu et al. fabricated sapphire FBGs via the femtosecond laser LBL technique, and a reflectivity of 6.3% was realized by optimizing fabrication parameters [15]. By inscribing an LBL-FBG with a reflectivity of 13.2% on an Yb-doped large core area double-clad fiber as a cavity mirror, a fiber laser with a spectral full width at a half-maximum of 0.56 nm was obtained [16]. With off-axis incidence and an LBL-inscribed few-mode FBG, an orbital angular momentum mode was achieved with high purity [17]. Despite various research on LBL-FBG applications being reported in the past decade, only a few of them discussed the fundamentality of LBL-FBGs. Although Huang et al. investigated the Fabry–Perot interference and Mach–Zehnder interference in the LBL-FBGs inscribed with a higher pulse energy (220 nJ) [18], the vacancy in the mechanism of LBL-FBG still needs to be filled. 

In this paper, we investigate the basic characteristics of LBL-FBGs dependent on the “line” position on the cross-plane of FBG. The cladding mode coupling is influenced by the “line” position, and LP1n cladding modes coupling becomes stronger when the “line” becomes far away from the center. Besides, by aligning all the lines across the core region, apodized line-by-line FBG can be realized. Our research work is believed to be meaningful in FBG inscription guidance.

## 2. Fabrication and Spectral Properties

**Figure 1:**
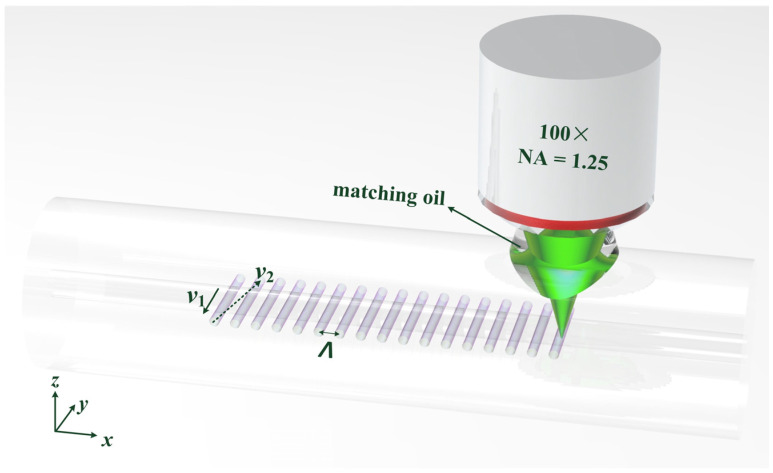
Inscription schematic of LBL-FBGs with oil-immersion lens.

The schematic setup of LBL-FBG inscription is demonstrated in Figure 1. A femtosecond laser (Pharos, Light-conversion) with a wavelength of 515 nm, a pulse width of 190 fs, and a repetition rate of 1 kHz was employed to inscribe LBL-FBGs. After passing through an oil-immersion lens with a magnification of 100× and an NA of 1.25, the femtosecond laser was focused in a specific region inside the fiber. Refractive index matching oil (n = 1.45) was used to eliminate aberrations induced by the fiber’s cylindrical shape. The pulse energy before the oil lens was about 36 nJ and the “line” width of an LBL-FBG was about 0.4 μm. The fiber used in our experiment was SMF28, which was fixed on a 3D electrical control translation stage (Newport (Irvine, USA): XMS160-S, XMS50-S and GTS30V). During inscription, the focus of the femtosecond laser was firstly translated along the y-axis at a speed of v1 = 500 μm/s, and the translation length was 32 μm, which is much longer than the diameter of core. Then, the focus translated to the beginning of the next “line” with a speed of v2 = 1180 μm/s. The diameter of the focus of the femtosecond laser is about 1μm and the diameter of each “point” is about 0.7 μm. The period (Λ) of FBG is defined as the interval between two successive lines. In our experiment, the period was fixed at 1070 nm, with which the second order Bragg resonance of LBL-FBG occurred around 1550 nm. For the convenience of cladding mode coupling investigation, fiber coating was stripped in our experiment.

**Figure 2:**
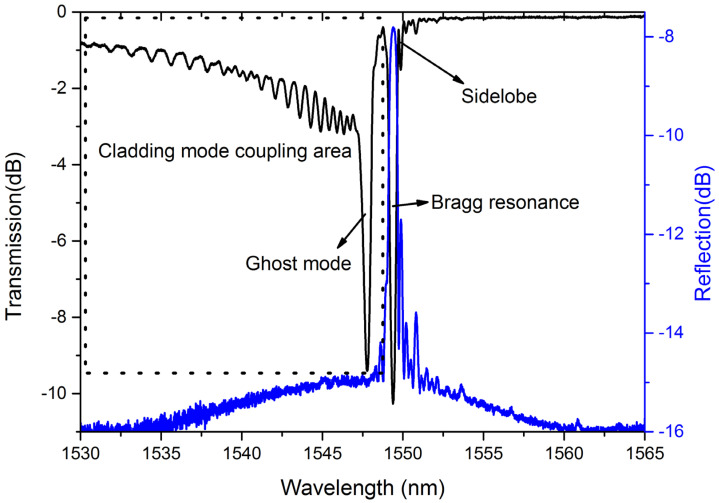
Typical spectrum of LBL-FBG (black-dotted-bordered rectangle represents the cladding mode coupling area).

Figure 2 performs the typical transmission and reflection spectrum of LBL-FBG. What has to be highlighted is that the spectra were measured by an optical spectrum analyzer (OSA) with a resolution of 0.05 nm and the LBL-FBG was immersed in refractive index matching oil during spectra measurement. Except Bragg resonance, cladding mode coupling can also be observed in the transmission spectrum and this phenomenon is much stronger than that in UV-exposed FBG [19]. A relatively stronger resonance occurs at a shorter wavelength than Bragg resonance, which is not present in the reflection spectrum. We infer that this resonance is ghost mode coupling, which occurs between core mode and several cladding modes. Besides, the sidelobe is clearly visible, especially in the reflection spectrum. In the rest of the paper, we will make a close study on the relationship between the basic characteristic of LBL-FBGs and the position of each “line”.

## 3. Cladding Mode Coupling in LBL-FBGs

**Figure 3:**
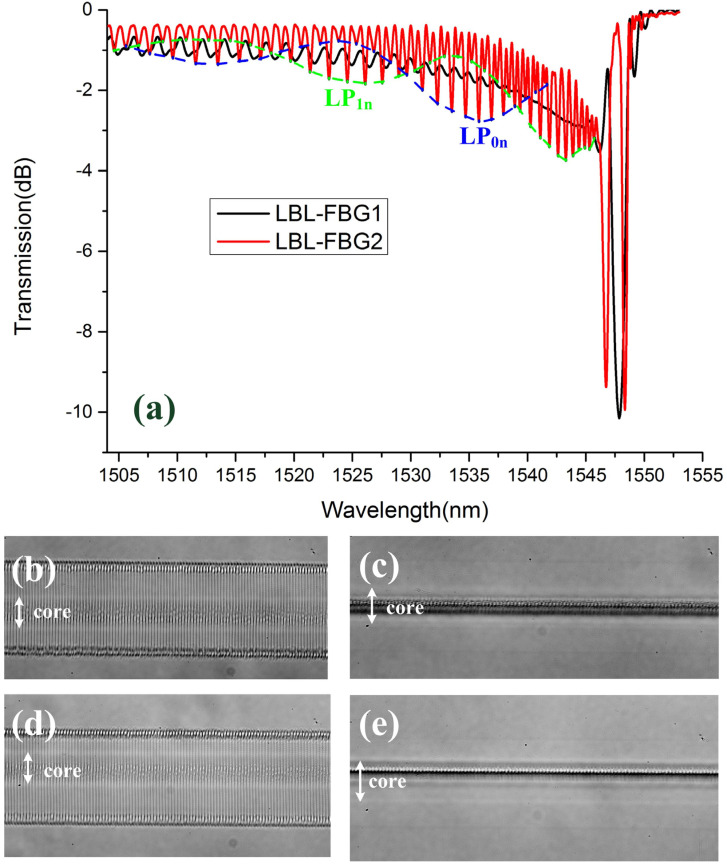
(**a**) Transmission spectrum of LBL-FBG1 and LBL-FBG2. Microscopy image (100×) of LBL-FBG1: (**b**) top view and (**c**) side view. Microscopy image (100×) of LBL-FBG2: (**d**) top view and (**e**) side view.

Figure 3 illustrates the transmission spectra and microscopy images (100×) of two different LBL-FBGs, namely LBL-FBG1 and LBL-FBG2. As the side view of LBL-FBG1 and LBL-FBG2 (Figure 3c,e) shows, LBL-FBG1 is located at the center of the core, but LBL-FBG2 is located near the edge between core and cladding; thus, the overlap between the fundamental mode and LBL-FBG1 was greater than that of LBL-FBG2. In order to ensure the same Bragg resonance intensity of these two FBGs, the length of LBL-FBG1 and LBL-FBG2 was 950.16 μm and 2643.97 μm, respectively. Figure 3a performs the transmission spectra of these two FBGs in air to avoid the influence of refractive index matching oil, and the spectra were recorded by a sweep wavelength system with a resolution of 3 pm. The shape of these two transmission spectra are completely different.

**Figure 4:**
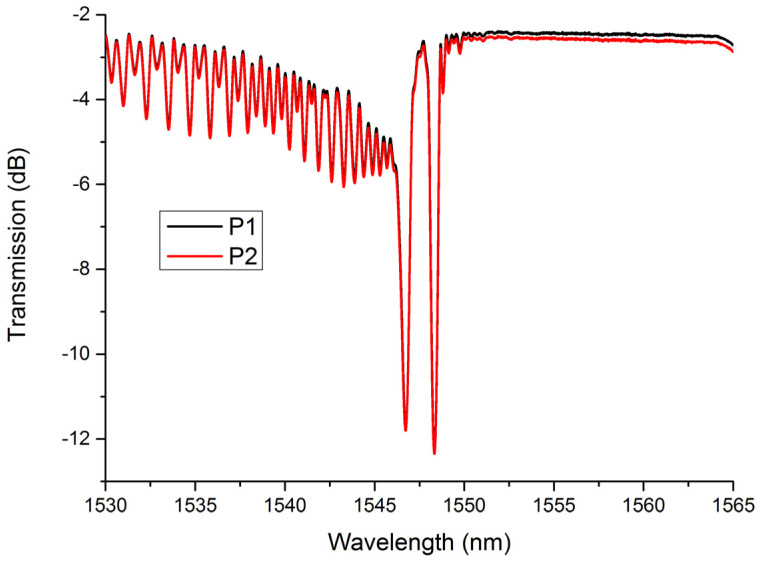
Transmission spectra of LBL-FBG2 under two different polarization states.

Beneficial from the inscription method, the polarization dependency of LBL-FBGs is extremely low. Figure 4 shows the transmission spectra of LBL-FBG2 under two different polarization states. In order to illustrate the polarization-dependent variation, we select two extreme conditions. It is very obvious that the polarization state exerts little effect on the transmission spectrum of LBL-FBG, even the grating located near the boundary between core and cladding. As the pulse energy was only 36 nJ, which is much lower than that in the literature [6,7], the refractive index profile in our experiment could be regarded as perturbation in the core region and the birefringence coefficient could not increase in this condition.

Firstly, the Bragg resonance bandwidth of these two FBGs is largely different. The bandwidth of LBL-FBG1 is wider than that of LBL-FBG2. To interpret this phenomenon, there needs to be focus on the fundamental mode of FBG. The theoretical transmission value in the Bragg wavelength is read as:

(1) $$T=1-\tanh^{2}(\kappa L)$$

where *κ* is the coupling coefficient of the fundamental mode and *L* is the length of grating. Due to the same transmission value (10 dB) as shown in Figure 3a, the value *κL* of these two gratings must be the same as well. The theoretical bandwidth is defined by:

(2) $$BW=\frac{\lambda^{2}}{\pi n_{eff}L}\left(\pi^{2}+{\left(\kappa L\right)}^{2}\right)$$

where *n_eff_* is the effective refractive index of the fundamental mode. From the above analysis, *κL* is a constant; hence, the bandwidth of grating is only affected by the length of grating (*L*). Since the length of LBL-FBG1 was much shorter than that of LBL-FBG2, the bandwidth of LBL-FBG1 was the wider one.

**Figure 5:**
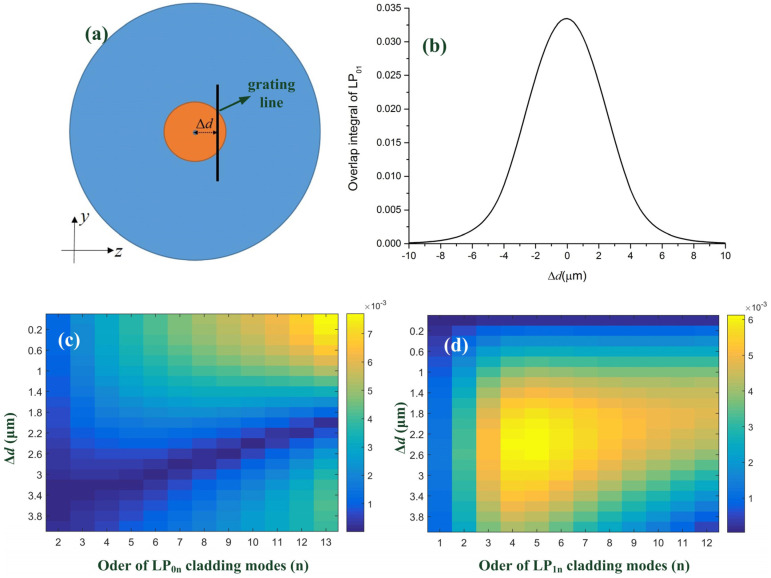
(**a**) Schematic of a cross-section of LBL-FBG. (**b**) Overlap integral of LP_01_ versus offset distance (Δ*d*). (**c**) Overlap integral of LP_0n_ cladding modes versus offset distance (Δ*d*). (**d**) Overlap integral of LP_1n_ cladding modes versus offset distance (Δ*d*).

The second difference between these two gratings is the cladding mode coupling. The cladding mode coupling of LBL-FBG2 performs the obvious grouping characteristics as the illustrated blue and green dash lines, which are not observed in LBL-FBG1. Besides, ghost mode resonance in LBL-FBG2 is much stronger than that of LBL-FBG1. We predict the cladding mode coupling in LBL-FBG is “line” position-dependent, just like what occurred in UV-exposed FBGs [20] and PBP-FBGs [6]. To test our prediction, the relevant model (Figure 5a), which presents the cross-section of LBL-FBG, is established to calculate the overlap integral between the fundamental mode and cladding mode. The vertical dark line represents the refractive index modulation induced by the femtosecond laser. The line width is set as 0.4 μm and the total length is 32 μm in order to mimic the situation in our experiment. Δ*d* in Figure 5a is defined as the offset distance (vertical distance) between the core center and the line. The resonance intensity between different modes is decided by the coupling coefficient:

(3) $$\kappa_{ab}=\frac{\pi c\varepsilon_{0}n_{core}}{\lambda }A_{ab}$$

where *c* is the light speed in vacuum, *ε*_0_ is the dielectric constant, *n_core_* is the refractive index of core, and *A_ab_* is the overlap integral between mode *a* and mode *b*, which is defined by:

(4) $$A_{ab}=\underset{core}{\iint }{\vec{\psi }}_{a}\left(x,y\right)\cdot \Delta n\left(x,y\right)\cdot {\vec{\psi }}_{b}^{*}\left(x,y\right)dxdy$$

where *ψ_a_*(*x*, *y*) and *ψ_b_*(*x*, *y*) are the normalized electric field of mode *a* and mode *b*, and Δ*n*(*x, y*) is the refractive index modulation induced by the femtosecond laser. For most situations, the refractive index modulation induced by femtosecond laser is in the 10^−3^ scale; thus, the value of Δ*n* is selected as 1×10^−3^ in this paper. Figure 5b demonstrates the overlap integral of LP_01_ mode. As the absolute value of Δ*d* increases, the overlap integral decreases and the overall tendency takes on a Gauss-like shape, which provides the potential for apodization of FBGs [21]. In [19], we found that, in UV-exposed FBGs, the fundamental mode mainly coupled to LP_0n_ and LP_1n_ cladding modes, for only these two series of cladding modes had stronger energy distribution in the core region. When it came to LBL-FBGs, the fundamental mode mainly transfers to these two series cladding modes as well. Considering the symmetry of fiber mode, we just need to study the situation where Δ*d* is above 0. Figure 5c illustrates the overlap integral between the fundamental mode and LP_0n_ cladding modes (we used a finite-element-method-based “three layers model” when we calculated cladding modes and no approximation was used), which decreases with increasing Δ*d*, because these cladding modes present Gauss-like intensity distribution in the core region just like LP_01_ mode. However, the overlap integral between the fundamental mode and LP_1n_ cladding modes shown in Figure 5d indicates that an increasing trend with Δ*d* for LP_1n_ cladding modes performs a doughnut-like intensity distribution in the core region, and the center of their mode profile is a phase singularity (light intensity is 0).

**Figure 6:**
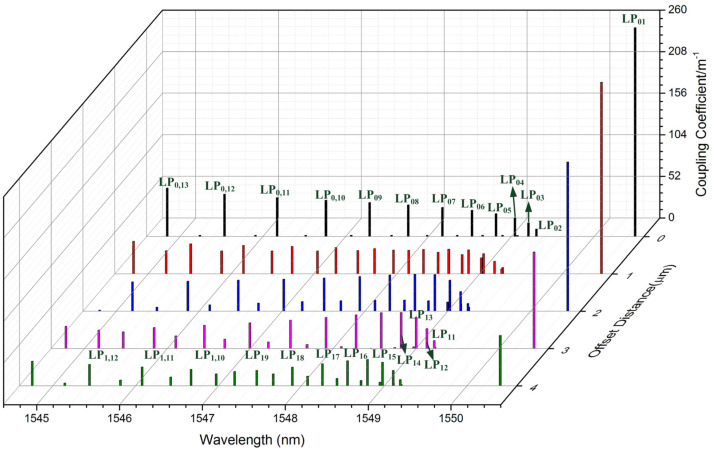
Coupling coefficients of LBL-FBG with different offset distance (Δ*d*).

Figure 6 demonstrates coupling coefficients of LBL-FBG with different Δ*d*. The simulation result agrees well with the experiment; the alternating resonance of LP_0n_ and LP_1n_ cladding modes decide two different envelopes in the transmission spectrum and these two envelopes overlap with each other. Moreover, the closer to the Bragg wavelength, the denser the cladding modes distribute, which contributed ghost mode resonance. When the “line” is located near the center of the core (the situation of LBL-FBG1), the fundamental mode mainly couples to LP_0n_ cladding modes, grouping characteristics of cladding mode coupling are not evident, and ghost mode resonance is not strong. However, when the “line” is located far from the core center (the situation of LBL-FBG2), LP_0n_ and LP_1n_ cladding modes oscillate simultaneously, and ghost mode resonance is clearly seen. Through the above analysis and the comparison between the transmission spectrum of LBL-FBG1 and LBL-FBG2, we can conclude that the blue dash line represents LP_0n_ cladding mode and the green dash line is LP_1n_ cladding mode. Besides, for the reason that the length of LBL-FBG2 is longer than that of LBL-FBG1, the cladding mode coupling of LBL-FBG2 is the stronger one. Different from off-core point-by-point inscription [6,7], the insertion loss and the polarization-dependent loss of off-core LBL-FBGs are extremely low, which provides potential usage in fiber lasers.

## 4. Apodized LBL-FBGs

If an LBL-FBG is intended to be applied in a sensor system, off-center writing will be a nice choice to fabricate a target FBG, which possesses a relatively thinner Bragg bandwidth and more abundant cladding mode coupling process. With those special characteristics, off-center LBL-FBGs are suitable for high precision and multichannel sensing applications. If an LBL-FBG aligns in the central plane of a fiber, the properties will be more stable. Therefore, this kind of LBL-FBGs may find applications in fiber laser systems.

**Figure 7:**
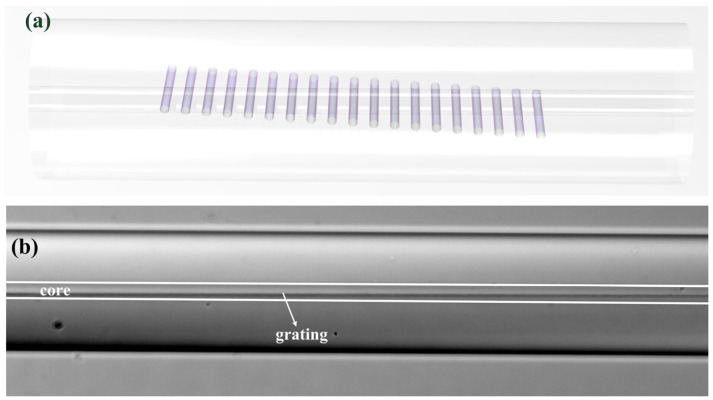
(**a**) Schematic of apodized LBL-FBG. (**b**) Microscopy (10×) of apodized LBL-FBG.

LBL-FBGs possess low insertion loss and low polarization-dependent loss and those characteristics make them a good candidate in high-power lasers. Before being applied in high-power lasers, some optimizations should be carried out to tailor spectrum properties of LBL-FBGs. In Figure 2, we highlighted that sidelobe was very obvious in both the reflection and transmission spectrum. Therefore, apodization of FBG is a necessary task. In [21], the authors reported that a Gaussian-apodized PBP-FBG could be realized by a linear translation of femtosecond laser focus across the fiber core in the process of grating fabrication. Fortunately, a similar method could also be utilized in LBL-FBGs. Figure 4b illustrates that different “line” positions decide different overlap integrals (self-coupling coefficient) of the fundamental mode. If all the lines align across the core, as in Figure 7a, the envelope of the self-coupling coefficient of the fundamental mode should be a Gaussian-like profile, just as Figure 5b shows. The side view of apodized LBL-FBG is indicated in Figure 7b. In order to obtain a large field of view, an objective with a magnification of 10 was utilized. Obviously, the apodized LBL-FBG was not limited in a plane parallel to the fiber axis. In total, 3001 lines constituted the apodized LBL-FBG (total length: 3210 μm), and the vertical distance between two successive lines was 5 nm; thus, about 2000 lines were located in the core region.

**Figure 8:**
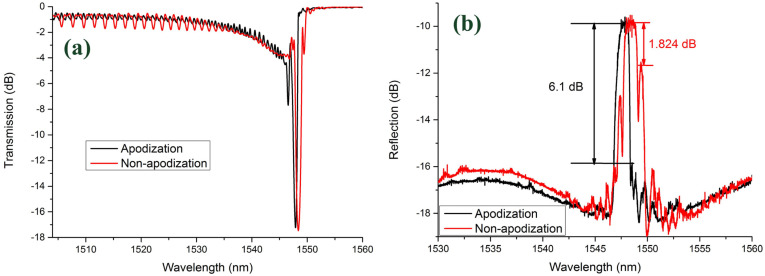
Comparison between apodized LBL-FBG and non-apodized LBL-FBG: (**a**) transmission spectrum and (**b**) reflection spectrum.

**Figure 9:**
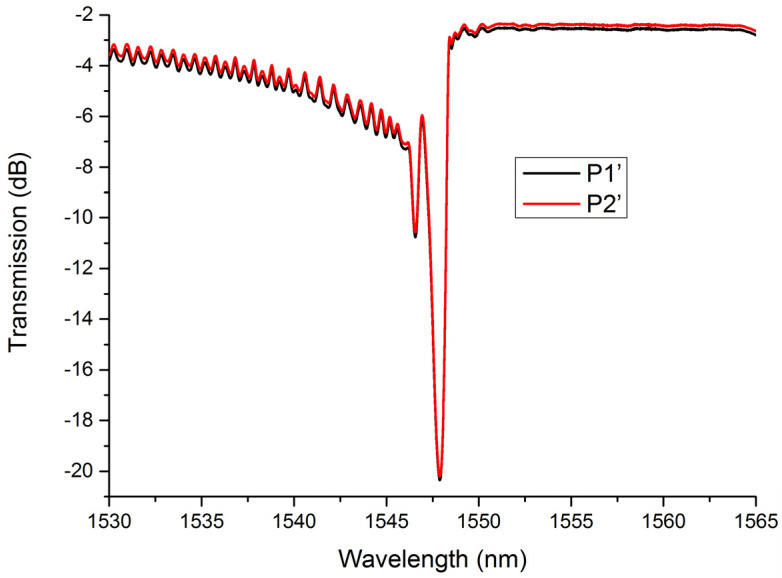
Transmission spectra of apodized LBL-FBG under different polarization states.

The transmission and reflection spectrum of the apodized LBL-FBG (measured in air with a high-resolution sweep frequency system) is given in Figure 8a,b. For comparison, a nonapodized LBL-FBG (length: 1319.31 μm) with the same resonance intensity (17 dB) as the apodized one was fabricated. With the proposed apodization method, cladding mode coupling could also be controlled. As Figure 8a shows, LP_0n_ and LP_1n_ cladding mode coupling occurs at the same time, but resonance intensity is extremely low compared with the nonapodized one, and the grouping characteristics are not observed. The sidelobe is evidently suppressed, as the reflection spectrum demonstrates. The Bragg peak to the first sidelobe was only 1.824 dB in the nonapodized LBL-FBG, but the value increased, to 6.1 dB in the apodized counterpart. The method proposed here is proved to be effective in apodization of FBG and cladding mode coupling can also be suppressed. This apodization method also does not induce any polarization dependency to the grating. Figure 9 illustrates the transmission spectra of the apodized LBL-FBG under two different polarization states (we select two extreme conditions). The birefringence effect is not obvious in this kind of situation and polarization state induces little effect on the apodized LBL-FBG.

## 5. Conclusions

In conclusion, we make a close study on the fundamental relationship between the cladding mode coupling properties of LBL-FBGs and the “line” position for the first time. Studies indicated that off-center writing resulted in more complex cladding mode coupling characteristics compared with a LBL-FBG with the same Bragg resonance intensity inscribed in the center. Apodized LBL-FBG was firstly realized by aligning all the lines across the fiber core. Our research works make up the vacancy in the fundamental mode of LBL-FBG and provides a guidance in LBL-FBG inscription. Femtosecond laser line-by-line inscription can realize high-quality FBGs with arbitrary resonant wavelength, which facilitates the application of FBGs in fiber oscillators. In the future, we will try to fabricate FBGs in rear earth-doped fiber using this method to realize monolithic fiber lasers.
